# Comparison of the operative and postoperative effects of del Nido and blood cardioplegia solutions in cardiopulmonary bypass surgery

**DOI:** 10.21470/1678-9741-2019-0436

**Published:** 2020

**Authors:** Yavuz Orak, Aydemir Kocarslan, Omer Faruk Boran, Mehmet Acıpayam, Erdinc Eroglu, Mehmet Kirisci, Adem Doganer

**Affiliations:** 1 Department of Anesthesiology and Reanimation, Faculty of Medicine, Kahramanmaras Sutcu Imam University, Kahramanmaras, Turkey.; 2 Department of Cardiovascular Surgery, Faculty of Medicine, Kahramanmaras Sutcu Imam University, Kahramanmaraş, Turkey.; 3 Department of Biostatistics and Medical Informatics, Faculty of Medicine, Kahramanmaras Sutcu Imam University, Kahramanmaraş, Turkey.

**Keywords:** Airway Extubation, Lactic Acid, Erythrocyte Indices, Glucose, Monocytes, Goals, Eosinophils, Heart Arrest, Induced, Cardiac Surgical Procedures, Blood Cell Count, Intensive Care Units

## Abstract

**Objective:**

Our goal was to compare the operative and postoperative effects of del Nido cardioplegia (DN group) and blood cardioplegia (BC group) performed in cardiac surgery.

**Methods:**

A total of 83 patients were included, separated into DN group and BC group. The operative and postoperative effects of the two groups were compared for the first 24 hours until extubation. The operative and postoperative complete blood count (CBC), biochemical values and clinical parameters were compared.

**Results:**

The first control activated clotting time (ACT) levels in DN group patients were lower (*P*=0.003) during the operation. The amount of cardioplegia in DN group were lower than that in BC group (*P*=0.001). The pump outflow and postoperative lactate level of DN group were lower than those of BC group (*P*=0.005, *P*=0.018, respectively), as well as the amounts of NaHCO3 (*P*=0.006) and KCl (*P*=0.001) used during the operation. The same occurred with the first monocytes (Mo) and mean corpuscular volume (MCV) levels in the postoperative intensive care unit (*P*=0.006, *P*=0.002). However, the first glucose level and the eosinophil (Eo) level were higher in DN group (*P*=0.011, *P*=0.047, respectively).

**Conclusion:**

In the operative evaluation, the amount of cardioplegia, the first ACT levels, the pump outflow lactate level and the amounts of NaHCO_3_ and KCl in DN group were lower. In postoperative evaluation, measured level of lactate, Mo and MCV in DN group were all lower; their glucose and Eo levels were higher.

**Table:** 

Abbreviations, acronyms & symbols				
**ACT**	**= Activated clotting time**	** **	**HIMS**	**= Hospital information management system**
**BC**	**= Blood cardioplegia**		**ICU**	**= Intensive care unit**
**BMI**	**= Body mass index**		**KCl**	**= Potassium chloride**
**CABG**	**= Coronary artery bypass grafting**		**LVEF**	**= Left ventricular ejection fraction**
**CBC**	**= Complete blood count**		**MAC**	**= Minimum alveolar concentration**
**CPB**	**= Cardiopulmonary bypass**		**MCV**	**= Mean corpuscular volume**
**DM**	**= Diabetes mellitus**		**Mo**	**= Monocytes**
**DN**	**= del Nido cardioplegia**		**MgSO_4_**	**= Magnesium sulphate**
**Eo**	**= Eosinophils**		**NaHCO_3_**	**= Sodium bicarbonate**
**FiO_2_**	**= Fraction of inspired oxygen**		**SPSS**	**= Statistical Package for the Social Sciences**

## INTRODUCTION

In cardiac surgery, the myocardial injury sustained during the operation is the most important cause of mortality and morbidity. The application of cardiopulmonary bypass (CPB) and elective cardiac arrest provided surgeons with the ability to operate in a blood-free environment and the precious time necessary for meticulous work. Ensuring adequate myocardial protection during the surgery, as well as before and after the operation, is the most significant factor of success^[1,2]^. Cardioplegia, as well as local and systemic hypothermia, have been used in cardiac surgery for many years for myocardial protection^[3,4]^. The causes of myocardial damage are global myocardial ischemia (aortic cross-clamp) and especially reperfusion^[5]^. Reperfusion following the ischemic period, although a definite need to maintain the viability of ischemic tissue, can itself cause serious or even potentially lethal damage to tissue that otherwise could have survived^[6]^. Cardioplegia, which is an elective and chemical cardiac arrest technique, was first applied as potassium cardioplegia in 1955 in cardiovascular surgery by Melrose^[7]^. Researchers at the University of Pittsburgh (Pittsburgh, PA) developed a new formulation for myocardial protection in the early 1990s. This team, led by Pedro del Nido, Hung Cao-Danh, K. Eric Sommers, and Akihiko Ohkado, patented this solution as a result^[8]^. Changes have been made to the original solution and, in the literature, its use in clinical practice is referred to as del Nido cardioplegia^[9]^.

Our goal in this study was to compare the effects of del Nido cardioplegia with those of blood cardioplegia, both during the operation and in the first 24 postoperative hours until extubation, in patients undergoing CPB with the mechanical ventilator turned off throughout the pumping period.

## METHODS

Approval for this study was granted (#2018/12-06) on July 13, 2018, by the Clinical Research Ethics Committee of the Faculty of Medicine, Kahramanmaras Sutcu Imam University. All patients included in the study had on-pump CPB between May 1, 2017, and August 15, 2018. Patient files and hospital information management system (HIMS) records were retrospectively reviewed and, of 150 cases operated on between these dates, 83 were included in the study.

Patients were divided into two groups: those who received del Nido cardioplegia (DN group, n=43) and those who received blood cardioplegia (BC group, n=40). Components of the solution used in el Nido and blood cardioplegia was shown in Table 1. All patients included in the study underwent non-emergency CPB with the mechanical ventilator turned off while on the pump, were extubated in the first 24 postoperative hours, and no patient suffered complications during the same 24-hour period. Exclusion criteria were emergency patients; patients who either were never on the pump or whose mechanical ventilators were on during CPB; patients who were extubated more than 24 hours after surgery; and patients with bleeding diathesis, renal failure, uncontrolled diabetes mellitus (DM), and electrolyte imbalance. Recorded information for each patient included their age; sex; body mass index (BMI); comorbid diseases; smoking/non-smoking status; CPB time; cross-clamping time; first measured activated clotting time (ACT) level; first amount of heparin administered and corresponding ACT level at the beginning of CPB; amount of protamine and corresponding ACT level; type and amount (mL) of cardioplegia used; lactate and glucose level at the beginning and at the end of CPB; length of surgery; and amounts of sodium bicarbonate (NaHCO_3_, 84 mg/mL, ampoule), potassium chloride (KCL, 7.5%, ampoule), magnesium sulphate (MgSO_4_, 15%, ampoule), Calcium Picken (calcium gluconate 10%), furosemide (20 mg/2 ml, ampoule), and 20% mannitol (100 ml) used during CPB. Complete blood count (CBC) and biochemical and albumin levels, the amount of urine produced prior to extubation and time of extubation were all recorded in the intensive care unit (ICU) for the first 24 hours after surgery. The use of operative and postoperative inotropic agents and the development of postoperative atrial fibrillation in the ICU were investigated.

**Table 1:** Components of the solution used in del Nido and blood cardioplegia.

Variables	Del Nido cardioplegia	Blood cardioplegia
**Components**		
NaCl (mEq)	0	77
KCl (mEq)	26	20
NaHCO_3_ (mEq)	13	10
20% mannitol (mL)	17	20
15% MgSO_4_ (mL)	14	10
2% lidocaine (mL)	6.5	0
Cardioplegia: blood ratio	4:01	

Acetate=27 mEq; Cl=98 mEq; gluconate=23 mEq; K=5 mEq; Mg=3 mEq; Na=141 mEq; pH=7.40; phosphate=1 mEq

Surgery was performed by two separate surgical teams at the cardiovascular surgery clinic. In all cases, antegrade cardioplegia was used and CPB with mild systemic hypothermia (30 to 34°C). Anesthesia was induced with 0.01 mg/kg of midazolam, 5 to 10 µg/kg of fentanyl, and 0.1 mg/kg of pancuronium. Pancuronium was administered continuously every half hour. Left radial artery cannulation was performed, followed by central venous catheterization. Sevoflurane 1-2 minimum alveolar concentration (MAC) was used in the operation. The mechanical ventilator settings were: FiO_2_ 0.5 or 50%, tidal volume 7 to 10 mL/kg, respiratory rate 12, inspiration/expiration rate 1/2, flow of 2 L/min.

### Statistical Analysis

Data were analyzed by the Shapiro-Wilk test, while the independent samples t-test was used to examine the differences between the BC and DN cardioplegia groups. Statistical parameters were expressed as mean±SEM. We examined the distribution of categorical variables according to groups using the chi-square test. In categorical variables, the statistical parameters were expressed as percentages (%) and frequencies (n). Statistical significance was accepted as *P*<0.05. Data were evaluated with IBM SPSS 22 software.

## RESULTS

The mean age of the patients in DN group was 54.95 years and those in BC group was 59.83 years. There were 22 male patients (51.16%) in DN group and 23 (57.50%) in BC group (Table 2).

**Table 2:** Sociodemographic characteristics.

			Groups		*P*-value
			Blood cardioplegia (n=40)	Del Nido cardioplegia (n=43)	
Age		Mean±SEM	59.83±2.49	54.95±2.57	0.178
BMI (kg/m^2^)		Mean±SEM	27.06±0.72	28.47±1.06	0.281
EF		Mean±SEM	56.5±2.1	55.3±2.4	0.704
Sex	Male	n (%)	23.00 (57.50)	22.00 (51.16)	0.553
	Female	n (%)	17.00 (42.50)	21.00 (48.84)	
Smoking	Smoker	n (%)	7.00 (17.50)	6.00 (14.29)	0.690
	Non-smoker	n (%)	33.00 (82.50)	36.00 (85.71)	
Number of years smoking		Mean±SEM	15.20±1.35	13.10±1.82	0.362

Chi-square test; Fisher's exact test; α=0.05; independent samples t-test. BMI=body mass index; EF=ejection fraction

Coronary artery bypass grafting (CABG) patients comprised 41.8% of the DN group members and 57.5% of the BC group. Among the comorbidities observed in patients, hypertension was quite high (Table 3).

**Table 3:** Distribution of diseases and comorbidities.

		Groups				*P*-value
		Blood cardioplegia		Del Nido cardioplegia		
		n=40	%	n=43	%	
Disorders	CABG	23	57.5	17	41.8	0.567
	AVR+MVR	3	7.5	4	9.3	
	AVR+CABG	1	2.5	3	7.0	
	AVR	3	7.5	2	4.7	
	VSD	1	2.5	2	4.7	
	MVR	4	10.0	4	9.3	
	ASD	5	12.5	4	9.3	
	MVR+CABG	0	0.0	2	4.7	
	Aortic aneurysm	0	0.0	2	4.7	
	TVR	0	0.0	1	2.3	
	MVR+TVR	0	0.0	1	2.3	
Additional diseases	Inguinal hernia	1	2.5	0	0.0	
	HT	18	45.0	19	44.2	
	DM	11	27.5	16	37.2	
	COPD	5	12.5	9	20.9	
	Hypothyroidism	3	7.5	4	9.3	
	CVD	3	7.5	5	11.6	
	Hyperthyroidism	0	0.0	2	4.7	
	Rheumatoid arthritis	0	0.0	1	2.3	

Fisher's exact test; α=0.05. ASD=atrial septal defect; AVR=aortic valve replacement; CABG=coronary artery bypass grafting; COPD=chronic obstructive pulmonary disease; CVD=cerebrovascular disease; DM=diabetes mellitus; HT=hypertension; MVR=mitral valve replacement; TVR=tricuspid valve replacement; VSD=ventricle septal defect

During the operation, the first ACT levels were lower in DN group patients than in BC group patients. The difference between them was statistically significant (*P*=0.003). The amounts of cardioplegia used were 955.81±32.78 mL in DN group and 1667.74±84.03 mL in BC group, and this difference was statistically significant (*P*=0.001). The pump outflow lactate level of the DN group was also lower by a statistically significant amount (*P*=0.005). The amount of NaHCO_3_ and KCl used during the surgeries were also lower for DN group patients, with statistically significant differences (*P*=0.006 and *P*=0.001, respectively) (Table 4). In the postoperative ICU, the first lactate level was lower in DN group by a statistically significant level (*P*=0.018). From the postoperative CBC tests, the first monocytes (Mo) and mean corpuscular volume (MCV) levels in the DN group were both low when compared to those in the BC group. The differences were statistically significant (*P*=0.006 and *P*=0.002, respectively). In the postoperative ICU, the first measured glucose level and eosinophil (Eo, %) level were higher in DN group, and this was statistically significant (P=0.011 and *P*=0.047, respectively) (Table 5). The number of patients who received noradrenaline during operation was higher in the DN group (*P*=0.013). There was no difference between the groups in terms of postoperative atrial fibrillation development (*P*=0.692) (Table 6).

**Table 4:** Distribution of operating parameters by type of cardioplegia.

	Groups		*P*-value
	Blood cardioplegia Mean±SEM	Del Nido cardioplegia	
		Mean±SEM	
CPB time (min)	112.43±6.40	114.58±6.30	0.811
Aortic cross-clamping time (min)	70.73±6.17	77.93±6.12	0.410
First measured ACT	122.85±3.73	106.79±3.64	0.003*
Heparin used at inflow (IU)	29712.50±1085.14	31511.63±1085.3	0.245
Inflow active coagulation time	588.10±29.98	671.74±33.56	0.068
Outflow active coagulation time	131.26±2.26	126.65±2.22	0.152
Prothrombin used at outflow (IU)	29565.79±1115.67	32418.60±1098.7	0.073
Amount of cardioplegia used (mL)	1667.74±84.03	955.81±32.78	0.001*
Inflow lactate (mmol/L)	1.4200±0.15	1.1405±0.08	0.110
Outflow lactate (mmol/L)	3.0136±0.21	2.3049±0.13	0.005*
Inflow glucose (mg/dL)	132.03±7.08	136.33±7.38	0.676
Outflow glucose (mg/dL)	198.61±6.40	202.21±5.76	0.676
Pump NaHCO_3_ (ampoule)	9.95±0.46	8.40±0.31	0.006*
Pump KCl (ampoule)	5.13±0.20	3.88±0.11	0.001*
Pump MgSO_4_ (ampoule)	3.05±0.13	3.12±0.11	0.712
Pump mannitol (ampoule)	106.25±5.714	104.65±3.24	0.805
Pump furosemide (ampoule)	1.25±0.06	1.14±0.05	0.207
Surgery time (min)	238.15±10.85	237.33±9.38	0.954

Independent samples t-test; α=0.05;

* Difference is statistically significant.

ACT=activated clotting time; CPB=cardiopulmonary bypass

**Table 5:** Distribution of the first biochemical and clinical parameters in the first 24 postoperative hours, by type of cardioplegia.

	Groups		*P*-value
	Blood cardioplegia Mean±SEM	Del Nido cardioplegia Mean±SEM	
Glucose (mg/dL)	172.97±8.86	203.79±7.67	0.011*
Lactate (mmol/L)	3.22±0.38	2.31±0.16	0.018*
Extubation time	404.25±31.33	447.98±32.38	0.335
Urine produced until extubation	1244.32±103.52	1395.71±82.04	0.250
Albumin (mg/dL)	3.24±0.08	3.37±0.07	0.262
pH	7.40±0.01	7.42±0.01	0.338
PO_2_	107.06±6.77	93.97±5.65	0.139
PCO_2_	35.14±0.91	35.81±0.86	0.596
Hg (g/dL)	10.88±0.22	10.42±0.19	0.111
Htc (%)	33.56±0.69	32.11±0.55	0.101
HCO_3_	22.69±0.30	23.16±0.41	0.371
K (mmol/L)	3.97±0.12	4.05±0.09	0.544
Na (mmol/L)	137.18±4.08	138.02±0.63	0.822
Ca (mg/dL)	1.27±0.20	1.06±0.03	0.270
WBC (10^9/L)	14.80±0.81	13.04±0.58	0.076
Ne (10^9/L)	12.60±0.75	11.13±0.52	0.105
Ne (%)	84.78±0.79	85.03±0.65	0.803
Ly (10^9/L)	0.90±0.08	0.92±0.09	0.833
Ly (%)	6.48±0.59	7.23±0.66	0.401
Mo (10^9/L)	1.28±0.10	0.95±0.06	0.006*
Mo (%)	8.63±0.54	7.38±0.41	0.067
RBC (10^6 U/L)	3.53±0.06	3.69±0.05	0.058
MCV (fL)	86.08±0.68	82.51±0.85	0.002*
MCH (pg)	30.11±1.60	27.47±0.36	0.095
RDW (fL)	44.46±0.68	43.66±0.62	0.388
Plt (10^9/L)	184531.25±11783.26	165875.00±7301.23	0.173
BUN (mg/dL)	32.44±2.92	26.23±.68	0.121
Creatinine (mg/dL)	1.31±0.14	1.24±0.14	0.731
AST (U/L)	78.13±10.07	69.89±6.39	0.484
ALT (U/L)	33.53±5.12	30.46±2.84	0.593
Magnesium (mg/dL)	2.39±0.08	2.36±0.09	0.763
Ba (10^9/L)	.01±0.00	0.06±0.05	0.347
Ba (%)	.06±0.01	0.06±0.01	0.910
Eo (10^9/L)	.01±0.00	0.03±0.01	0.064
Eo (%)	.07±0.03	0.29±0.10	0.047*
RDW (%)	14.66±0.23	15.11±0.31	0.253

Independent samples t-test; α=0.05;

* Difference is statistically significant.

ALT=alanine aminotransferase; AST=aspartate aminotransferase; Ba=basophils; BUN=blood urea nitrogen; Ca=calcium; Eo=eosinophils; HCO_3_=bicarbonate; Hg=hemoglobin; Htc=hematocrit; K=potassium; Ly=lymphocytes; MCH=mean corpuscular hemoglobin; MCV=mean corpuscular volume; Mo=monocytes; Na=sodium; Ne=neutrophils; PCO_2_=partial pressure of carbon dioxide; Plt=platelets; PO_2_=partial pressure of oxygen; RBC=red blood cells; RDW=red blood cell distribution width; WBC=white blood cells

**Table 6:** Use of inotropic agent and development of postoperative atrial fibrillation by groups.

		Groups				*P*-value
		Blood cardioplegia		Del Nido cardioplegia		
		(n=40)	%	(n=43)	%	
Operative dopamine	Yes	36	90.0	41	95.3	0.347
	No	4	10.0	2	4.7	
Operative dobutamine	Yes	5	12.5	2	4.7	0.199
	No	35	87.5	41	95.3	
Operative noradrenaline	Yes	29	72.5	40	93.0	0.013*
	No	11	27.5	3	7.0	
Operative adrenaline	Yes	1	2.5	1	2.3	0.959
	No	39	97.5	42	97.7	
Postoperative dopamine	Yes	25	62.5	28	65.2	0.693
	No	15	37.5	15	34.8	
Postoperative noradrenaline	Yes	9	22.5	10	23.3	0.935
	No	31	77.5	33	76.7	
Postoperative adrenaline	Yes	1	2.5	0	0.0	-
	No	39	97.5	43	100.0	
Postoperative dobutamine	Yes	0	0.0	0	0.0	-
	No	40	100.0	43	100.0	
Postoperative atrial fibrillation	Yes	7	17.5	9	20.9	0.692
	No	33	82.5	34	79.1	

Chi-square test; Fisher's exact test; a=0.05.

* Difference is statistically significant.

## DISCUSSION

In the operative evaluation of our study, the amount of cardioplegia (mL), the first measured ACT values, the outflow lactate level, the amounts of NaHCO_3_ used, and the amounts of KCl in DN group were all lower. In the postoperative ICU evaluation, first lactate level, Mo, and MCV levels in DN group were all lower and the first glucose levels and Eo were higher than those of BC group.

In studies similar to ours, a smaller amount of cardioplegia (mL) were used in DN group^[10,11]^. In another study, comparing the use of del Nido with that of St. Thomas’ cardioplegic solution, it was shown that del Nido cardioplegia resulted in shorter cross-clamp and CPB times, less cardioplegia used, and better myocardial protection in terms of left ventricular ejection fraction (LVEF)^[12]^. The CPB times in our study were shorter for BC group than for DN group, but this difference was not statistically significant. In a separate study, cardiac ischemia and total CPB times were shorter in DN group than in BC group. This difference is probably attributable to the normothermic strategy used in the DN group and the feasibility of the single-dose infusion^[13]^. Del Nido cardioplegia has been shown to be safe in the reoperative aortic valve^[11]^ and post-infarction CABG surgeries^[14]^. It has also been reported not only to reduce operative time but also to result in myocardial protection and cost-effectiveness in isolated valve surgeries^[15]^. In the postoperative ICU evaluation of our study, glucose levels in DN group were higher than those of BC group. A different study reported that the myocardial protection and clinical results provided by del Nido cardioplegia in adult isolated CABG patients is equal to that provided by blood cardioplegia, while the CPB glucose levels of del Nido are lower than those of blood cardioplegia^[16]^. There was no difference in the operative blood glucose level in our study.

Del Nido solution is safe for use in adult primary isolated aortic and mitral valve operations. Some surgical approaches have the advantage of reducing surgical time, cost, postoperative insulin need, surgical downtime, and also give a more positive outcome with regard to blood glucose levels^[16]^. Myocardial injury measurements, such as troponin T levels, postoperative LVEF, and postoperative inotropic/vasopressor support, were similar to those in patients who received Buckberg solution. The crystalloid component of the del Nido solution is not glucose-based (unlike the Buckberg solution); patients who received the del Nido solution had lower blood sugar levels and needed less insulin in the postoperative period^[15]^. In our study, the number of patients using noradrenaline during the operation was higher in the DN group. But there was no difference between groups in terms of postoperative inotropic use. In addition, there was no difference between the two groups in terms of the development of postoperative atrial fibrillation. Del Nido cardioplegia has been shown to be associated with reduced spontaneous activity and myocardial injury, and with better functional recovery during arrest in isolated older hearts, when compared to blood cardioplegia^[17]^. In one study, del Nido cardioplegia solution had the potential to provide superior myocardial protection in elderly hearts by preventing electromechanical activity during cardioplegic arrest and Ca^2+^-induced hypercontraction during early reperfusion^[18]^. Studies have shown that the sustained release of lactate or higher lactate levels in the reperfusion period after CABG is not satisfactory for myocardial protection and the improvement of aerobic metabolism decreases perioperatively^[19]^.

In our study, the pump outflow and postoperative lactate levels in DN group were lower than in BC group. The low operative and postoperative lactate levels in DN group, and its smaller amount of cardioplegia, were found to be advantageous in terms of myocardial protection and clinical follow-up. Since the amounts of NaHCO_3_ and KCL used were higher in DN group, the low amounts of NaHCO_3_ and KCL used throughout the operation were considered normal.

In the postoperative ICU evaluation, the lower the first measured Mo and MCV values, the higher the first glucose level, which can be seen as a disadvantage of del Nido cardioplegia. It is our belief that our study will constitute an example in the literature and lead to new studies. The limitations of our study are its retrospective nature and the diversity of cases.

## CONCLUSION

In our study, the operative and postoperative effects in DN group and BC group were investigated. In the operative evaluation, the cardioplegic amount (mL) used and the pump outflow lactate level in DN group were lower. This was an advantage in terms of myocardial protection and clinical follow-up. In the postoperative evaluation in the ICU, the first measured level of lactate, Mo, and MCV in DN group were all lower, while the first glucose level was higher. The low postoperative lactate level was considered an advantage of del Nido cardioplegia, whereas the lower the Mo and MCV values, the high first glucose level, which was seen as a disadvantage. These results will bring different perspectives on the use of del Nido and blood cardioplegia and will pave the way for new studies.

**Table:** 

Authors' roles & responsibilities	
YO	Substantial contributions to the conception or design of the work; or the acquisition, analysis, or interpretation of data for the work; drafting the work or revising it critically for important intellectual content; agreement to be accountable for all aspects of the work in ensuring that questions related to the accuracy or integrity of any part of the work are appropriately investigated and resolved; final approval of the version to be published
AK	Drafting the work or revising it critically for important intellectual content; final approval of the version to be published
OFB	Agreement to be accountable for all aspects of the work in ensuring that questions related to the accuracy or integrity of any part of the work are appropriately investigated and resolved; drafting the work or revising it critically for important intellectual content; final approval of the version to be published
MA	Substantial contributions to the conception or design of the work; or the acquisition, analysis, or interpretation of data for the work; final approval of the version to be published
EE	Substantial contributions to the conception or design of the work; or the acquisition, analysis, or interpretation of data for the work; final approval of the version to be published
MK	Agreement to be accountable for all aspects of the work in ensuring that questions related to the accuracy or integrity of any part of the work are appropriately investigated and resolved; drafting the work or revising it critically for important intellectual content; final approval of the version to be published
AD	Substantial contributions to the conception or design of the work; or the acquisition, analysis, or interpretation of data for the work; agreement to be accountable for all aspects of the work in ensuring that questions related to the accuracy or integrity of any part of the work are appropriately investigated and resolved; final approval of the version to be published

## References

[1] Özdöl Ç, Erol Ç, Paç M, Akçevin A, Aka SA, Büket S, Sarioglu T (2013). Kalp cerrahisinde miyokard korumasi. Kalp ve Damar Cerrahisi, I. Cilt 2.

[2] Skubas N, Lichtman AD, Sharma A, Thomas SJ, Barash PG, Cullen BF, Stoelting RK, Calahan MK, Stock MC, Günaydin B, Demirkan O (2012). Kalp cerrahisinde anestezi. KlinikAnestezi 5.

[3] Roe BB, Hutchinson JC, Fishman NH, Ullyot DJ, Smith DL (1977). Myocardial protection with cold, ischemic, potassium-induced cardioplegia. J Thorac Cardiovasc Surg.

[4] Conti VR, Bertranou EG, Blackstone EH, Kirklin JW, Digerness SB (1978). Cold cardioplegia versus hypothermia for myocardial protection. Randomized clinical study. J Thorac Cardiovasc Surg.

[5] Buckberg GD (1987). Strategies and logic of cardioplegic delivery to prevent, avoid, and reverse ischemic and reperfusion damage. J Thorac Cardiovasc Surg.

[6] Hearse DJ (1990). Ischemia, reperfusion, and the determinants of tissue injury. Cardiovasc Drugs Ther.

[7] Melrose DG, Dreyer B, Bentall HH, Baker JB (1955). Elective cardiac arrest. Lancet.

[8] Del Nido PJ, Cao-Danh H, Sommers KE, Ohkado A, University of Pittsburgh of the Commonwealth System of Higher Education An aqueous heart preservation and cardioplegia solution.

[9] Matte GS, del Nido PJ (2012). History and use of del Nido cardioplegia solution at Boston children’s hospital. J Extra Corpor Technol.

[10] Ad N, Holmes SD, Massimiano PS, Rongione AJ, Fornaresio LM, Fitzgerald D (2018). The use of del Nido cardioplegia in adult cardiac surgery: a prospective randomized trial. J Thorac Cardiovasc Surg.

[11] Sorabella RA, Akashi H, Yerebakan H, Najjar M, Mannan A, Williams MR (2014). Myocardial protection using del nido cardioplegia solution in adult reoperative aortic valve surgery. J Card Surg.

[12] Mishra P, Jadhav RB, Mohapatra CK, Khandekar J, Raut C, Ammannaya GK (2016). Comparison of del Nido cardioplegia and St. Thomas hospital solution - two types of cardioplegia in adult cardiac surgery. Kardiochir Torakochirur Pol.

[13] Kim WK, Kim HR, Kim JB, Jung SH, Choo SJ, Chung CH (2018). del Nido cardioplegia in adult cardiac surgery: beyond single-valve surgery. Interact Cardiovasc Thorac Surg.

[14] Yerebakan H, Sorabella RA, Najjar M, Castillero E, Mongero L, Beck J (2014). Del Nido Cardioplegia can be safely administered in high-risk coronary artery bypass grafting surgery after acute myocardial infarction: a propensity matched comparison. J Cardiothorac Surg.

[15] Mick SL, Robich MP, Houghtaling PL, Gillinov AM, Soltesz EG, Johnston DR (2015). del Nido versus Buckberg cardioplegia in adult isolated valve surgery. J Thorac Cardiovasc Surg.

[16] Timek T, Willekes C, Hulme O, Himelhoch B, Nadeau D, Borgman A (2016). Propensity matched analysis of del Nido cardioplegia in adult coronary artery bypass grafting: initial experience with 100 consecutive patients. Ann Thorac Surg.

[17] Govindapillai A, Hua R, Rose R, Friesen CH, O’Blenes SB (2013). Protecting the aged heart during cardiac surgery: use of del Nido cardioplegia provides superior functional recovery in isolated hearts. J Thorac Cardiovasc Surg.

[18] O’Blenes SB, Friesen CH, Ali A, Howlett S (2011). Protecting the aged heart during cardiac surgery: the potential benefits of del Nido cardioplegia. J Thorac Cardiovasc Surg.

[19] Kurt MI, Yurtseven N, Tuygun AK, Savaskan D, Canik S (2010). Myocardial protection: standard versus insulin cardioplegia and glucose-insulin-potassium solutions. Turkish J Thorac Cardiovasc Surg.

